# Isolated Gallbladder Rupture Due to Blunt Abdominal Trauma

**DOI:** 10.1155/1989/95937

**Published:** 1989

**Authors:** P. Schachter, A. Czerniak, E. Shemesh, I. Avigad, G. Lotan, I. Wolfstein

**Affiliations:** 1 From the Departments of Surgery and Gastroenterology the Chaim Sheba Medical Center Tel-Hashomer and Sackler School of Medicine Tel-Avil University Israel; 2 Department of Surgery “D” Chaim Seba Medical Center Israel

## Abstract

Traumatic injury to the extrahepatic biliary system is rare and usually diagnosed at laparotomy when it is associated with other visceral injuries. Isolated gallbladder rupture due to blunt abdominal trauma is even rarer. The clinical presentation of gallbladder injury is variable, resulting in a delay in diagnosis and treatment. Awareness to the possibilty of trauma to the extrahepatic biliary system enables early surgical intervention and eliminates the high morbidity associated with delated diagnosis.

A 5 year old child with isolated gallbladder rupture caused by blunt abdominal trauma is presented.


*HPB Surgery* 1989, Vol. 1, pp. 359–362
Reprints available directly from the publisher
Photocopying permitted by license only

© 1989 Harwood Academic Publishers GmbH
Printed in Great Britain

### CASE REPORT
### ISOLATED GALLBLADDER RUPTURE DUE TO BLUNT ABDOMINAL TRAUMA

P. SCHACHTER, A. CZERNIAK, E. SHEMESH, I. AVIGAD, G. LOTAN and I. WOLFSTEIN

*From the Departments of Surgery and Gastroenterology*, the Chaim Sheba Medical Center, Tel-Hashomer and Sackler School of Medicine, Tel-Avil University, Israel*

*(Received 25 April, 1988)*

Traumatic injury to the extrahepatic biliary system is rare and usually diagnosed at laparotomy when it is associated with other visceral injuries. Isolated gallbladder rupture due to blunt abdominal trauma is even rarer. The clinical presentation of gallbladder injury is variable, resulting in a delay in diagnosis and treatment. Awareness to the possibilty of trauma to the extrahepatic biliary system enables early surgical intervention and eliminates the high morbidity associated with delated diagnosis.

A 5 year old child with isolated gallbladder rupture caused by blunt abdominal trauma is presented.

KEY WORDS: Gallbladder rupture, trauma, blunt, gallbladder

### INTRODUCTION

The gallbladder is a well protected organ, being partially embedded in the liver substance and covered by the rib cage. Consequently, gallbladder rupture due to blunt trauma is rare, and usually associated with additional visceral injuries. Isolatedgallbladder rupture in pediatric patients following non-penetrating abdominal trauma is even rarer, causes subtle signs, resulting in delayed diagnosis and treatment. Early explorative laparotomy is recommended to reduce the high morbidity associated with this condition.

### *Case Report*

A 5-year-old boy was admitted to the Pediatric Surgical Service 4 hours after falling to the ground from a 5 meter high balcony.

On admission, he was haemodynamically stable with blood pressure of 140/100 mm Hg. and pulse rate of 100/min. Physical examination was essentially normal apart from tenderness over the right upper abdomen with hypoactive bowel sounds. Laboratory data including serum haemoglobin, haematocrit, glucose, amylase, electrolytes and urine examination were all within normal limits. The white cell count was 18,400/mm$^3$. Radiological studies of the skull, cervical spine, chest and abdomen x-rays (supine and erect) were normal.

Over the next hours, though he remained hemodynamically stable, his abdominal condition deteriorated, with the development of guarding and rigidity over the upper abdomen, necessitating an exploratory laparotomy, which was performed 3 hours

Correspondence to: Professor I. Wolfstein, M.D., Department of Surgery "D", Chaim Seba Medical Center, Israel.

after admission. At laparotomy, blood and bile were found in the infrahepatic region. The gallbladder was collapsed and avulsed from its hepatic bed with a 3cm. longitudinal tear on its posterior aspect (Figure 1). No other abdominal injuries were noted.

A cholecystectomy was performed and intraoperative cholangiography demonstrated a normal extrahepatic biliary tract.

Postoperative course was uneventful and the patient was discharged on the seventh postoperative day.

### DISCUSSION

Gallbladder injury due to blunt abdominal trauma is rare, with an incidence of about 2% of all intraabdominal injuries[1,2,3]. Liver injury is present in up to 83% of cases of gallbladder trauma, and the spleen, stomach, intestine, pancreas, and kidney may also be injured[3].

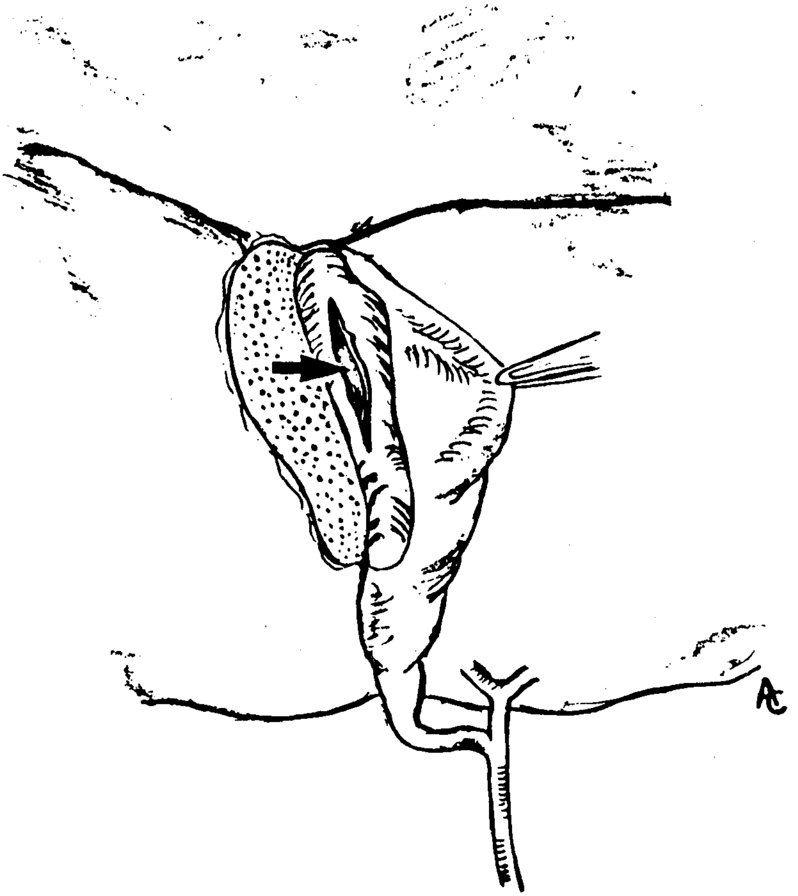

**Figure 1** Traumatic partial avulsion of the gallbladder from the gallbladder bed with rupture of the posterior gallbladder wall (arrow).

Isolated gallbladder rupture is of rare occurance. Review of the English literature since 1900 disclosed 51 such cases[1–18], ranging in age from 3 years[4] to 73 years[2], with about 30% of cases in the pediatric age group. Most injuries followed road accidents or direct blows to the abdomen.

Penn et al classified four major groups of gallbladder injury: contusion, avulsion, laceration and traumatic cholecystitis[1]. Delayed perforation following blunt abdominal trauma can also occur, following a hematoma of the gallbladder wall developing into an area of necrosis, or a blood clot occluding the cystic duct and precipitating infection, gangrene and late perforation[1,2].

A thin-walled normal gallbladder or a distended gallbladder following a meal is more prone to traumatic rupture than a diseased, thick walled one[17]. Indeed, only two cases with a ruptured gallbladder containing stones have been reported[2]. It is postulated that gallbladder distention at the time of trauma is a prerequisite for its rupture. A high incidence of traumatic gallbladder rupture is reported in alcohol intoxicated patients. Alcohol intake enhances gastrin and secretin secretion, which in turn stimulate bile flow and high serum level of alcohol elevates the common bile duct pressure by increasing the sphincter of Oddi's tone[13]. This combined effect causes gallbladder distension which makes it more susceptible to injury, especially since alcohol also relaxes the abdominal wall musculature[19].

Though persistent bile leakage causes sequestration of fluids resulting in the intraperitoneal bilious collections[1,2,9], the clinical presentation is variable and non-specific, resulting in a delay in diagnosis[20,21]. Why some patients tolerate intraperitoneal bile leak without short term sequelae, while others develop bile peritonitis, is a mystery[22]. Both conditions, however, require aggressive surgical intervention[13,20], since continuous bile loss into the peritoneal or retroperitoneal space usually has a fatal outcome[23].

Diagnostic procedures are not always helpful. Peritoneal tap or lavage is significant only when it yields bile-stained fluid, yet a high incidence of false-negative results has been reported[9]. Contrast x-rays studies depend upon the ability of the gallbladder to concentrate contrast media which may be disturbed in an injured gallbladder. Ultrasonography and computerized tomography scans are valuable when the bile leak becomes encapsulated yielding a mass, while in the acute phase their contribution is questionable[14]. Tc-99m-HIDA cholecystoscintigraphy was advocated by Ryan[24] as an accurate means of detecting bile leak. Recently, endoscopic retrograde cholangiography and percutaneous transhepatic cholangiography have been proven useful and accurate in diagnosing bile leakage[13,25], but their use as an emergency procedure in a trauma patient in questionable. Despite the availability of diagnostic methods, most reported patients with isolated injury to the extrahepatic biliary system were operated after a considerable delay and without preoperative accurate diagnosis. The need, therefore, for careful sequential clinical monitoring of patients with blunt trauma is reemphasized.

Cholecystorrhaphy or cholecystostomy have previously been suggested for the management of a ruptured gallbladder[6,7], but cholecystectomy is now accepted as the treatment of choice[2]. Morbidity of patients with gallbladder injuries is usually due to associated intraabdominal injuries[2] with an overall mortality between 3.8 and 16%[17]. However, no death has been reported in patients with isolated gallbladder rupture treated surgically.

Bearing in mind the possibility of injury to the extrahepatic biliary system and using the modern diagnostic facilities, particularly Tc-HIDA scintigraphy, prompt diagnosis and treatment may be possible, eliminating the high morbidity associated with delayed diagnosis.

### *References*

1. Penn, I. (1966) Injuries to the gallbladder. *Br. J. Surg.*, **49**, 636–641
2. Wiener, I., Watson, L.C. and Wolma, F.J. (1982) Perforation of the gallbladder due to blunt abdominal trauma. *Arch. Surg.* **117**, 805–807
3. Soderstrom, C.A., Maekawa, K., Du Priest, R.W. Jr. and Cowley, R.A. (1981) Gallbladder injuries resulting from blunt abdominal trauma. *Ann. Surg.* **193**, 60–66
4. Benson, C.D. and Prust, F.W. (1953) Traumatic injuries of the liver, gallbladder and biliary tract in the infact and child. *Surg. Clin. North Am.*, **33**, 1187–1191
5. Norgore, M.N. (1946) Traumatic rupture of the gallbladder. *Ann. Surg.* **123**, 127–134
6. Hicks, J.A. (1944) Case of traumatic perforation of the gallbladder in a child of three years. *Br. J. Surg.* **31**, 305–306
7. Sengstacken, R.F. (1944) Traumatic rupture of the gallbladder. *Ann. Surg.* **119**, 959
8. Hartman, S.W. and Greaney, E.M. (1964) Traumatic injuries to the biliary system in children. *Am. J. Surg.* **108**, 150–154
9. Schechter, D.C. (1966) Solitary wounding of gallbladder from blunt abdominal trauma. *NY State J. Med.* **69**, 2895–2901
10. Evans, J.P. (1976) Traumatic rupture of the gallbladder in a three-year-old boy. *J. Pediat. Surg.* **11** 1033–1034
11. Brismar, B. (1977) Traumatic rupture of the gallbladder. *Acta Chir. Scand.* **143**, 377–378
12. Kneppel, P.A. and Riddel, R.V. (1956) Traumatic rupture of the gallbladder without a penetrating wound of the abdominal wall. *Arch. Surg.* **73**, 371–372
13. Spigos, D.G., Tan, W.S., Larson, G., Palani, C., Laintoon, M.M. and Capek, V. (1981) Diagnosis of traumatic rupture of the gallbladder. *Am. J. Surg.* **141**, 731–735
14. Gottesman, L., Marks, R.A., Khoury, P.T., Moallem, A.G. and Wichern, W.A. Jr. (1984) Diagnosis of isolated perforation of the gallbladder following blunt trauma using sonography and CT scan. *J. of Trauma* **24**(3), 280–281
15. Kaehr, D., Jones, L.M., Miller, S.F. and Finley, R.K. (1984) Traumatic cholecystectomy. *J. Trauma,* **24**, 544–545
16. Lineaweaver, W., Robertson, J. and Rumley, T. (1985) PIPIDA scan diagnosis of traumatic rupture of the gallbladder. *Injury* **16**, 238–240
17. Posner, M.C. and Moor, E.E. (1985) Extrahepatic biliary tract injury: operative management plan. *J. Trauma* **25**, 833–837
18. Greenwald, G., Stine, R.J. and Larson, R.E (1987) Perforation of the gallbladder following blunt abdominal trauma. *Ann. Emerg. Med.* **16**, 452–454
19. Smith, S.W. and Hastings, T.N. (1954) Traumatic rupture of the gallbladder. *Ann. Surg.* **139**, 517–520
20. Ackerman, N.B., Sillin, L.F. and Suresh, K. (1985) Consequences of intraperitoneal bile-bile ascites versus bile peritonitis. *Am. J. Surg.* **149**, 244–246
21. Frank, D.J., Pereiras, R., Souza-Lima, M.S., Taub, S.J. and Schiff, E.R. (1978) Traumatic rupture of the gallbladder with massive biliary ascites. *JAMA* **240**, 252–253
22. Blumgart, L.H. (1988) Surgery of the liver and biliary tract. Edinburgh, Churchill Livingstone
23. Feliciano, D.V., Bitondo, C.G., Burch, J.M., Mattox, K.L., Beall, A.C. and Jordan, G.L. (1985) Management of transhepatic injuries to the extrahepatic biliary ducts. *Am. J. Surg.* **1150**, 705–709
24. Ryan, J., Cooper, M., Loberg, M., Harvey, E. and Dikorski, S. (1977) Technetium 99m labeled N-iminodiacetic acid (Tc 99m HIDA): A new radiopharmaceutical for hepatobiliary imaging studies. *J. Nucl. Med.* **18**, 997–1004
25. Bill, K. and Belber, J.P. (1978) Diagnosis of gangrene and perforation of the gallbladder by endoscopic retrograde cholangiography. *A.J.R.* **130**, 67–70

